# Cognition, culture, and credibility: deconstructing feedback in medical education

**DOI:** 10.1007/s40037-014-0115-2

**Published:** 2014-03-07

**Authors:** Christopher Watling

**Affiliations:** Postgraduate Medical Education, Schulich School of Medicine and Dentistry, Western University, Medical Sciences Building Room M103, London, ON N6A 5C1 Canada

Why does some feedback become profoundly influential for learners, while other feedback is discarded as meaningless? What insights can other learning cultures offer to medicine about feedback? This thesis explores these questions, enriching our understanding of how individuals and the environment in which they learn interact to shape feedback’s impact.

## Introduction

Although feedback has been widely endorsed as an essential facilitator of learning [1–4], there have been few systematic investigations of how it becomes meaningful for learners. In this thesis, we address this important gap through a series of studies exploring feedback in the medical education setting. Defining feedback broadly as *information about a learner’s performance*, and drawing on the perspectives and reflections of those who have experienced it both within and outside of medical training, we begin to unravel the complex interplay of individual and sociocultural influences that shape the meaning of feedback for medical learners.

## Methods

The research questions posed in this thesis are exploratory, examining *how* and *why* feedback may or may not impact learners within the specific context of medical training. Exploratory questions may benefit from a qualitative approach [5]; accordingly, the five empiric studies comprising this thesis used a constructivist grounded theory methodology. Rooted in an interpretive tradition, constructivist grounded theory offers a rigorous approach to the qualitative study of social or social psychological processes [6–8].

Our first two studies approached the problem of feedback from the perspective of the individual learner. In our first study, we interviewed physicians new in practice, asking them to reflect on the experiences they had considered influential during their training. Constant comparative analysis to identify themes was conducted iteratively, with the goal of deriving a model of clinical learning and understanding the place of feedback within that model. In our second study, we set out to test the utility of Kluger and Van Dijk’s proposal that regulatory focus theory could enable a better understanding of how learners respond to feedback, and thus how feedback becomes (or fails to become) influential [9]. Regulatory focus theory posits two systems of self-regulation underlying human motivation: promotion focus, concerned with aspirations and accomplishments, and prevention focus, concerned with responsibilities and safety [10]. Using the lens of regulatory focus, we re-analysed all the instances in the data collected for our first study in which the experience of receiving and responding to feedback was described.

In our third, fourth, and fifth studies, we explored feedback from a sociocultural perspective. In our third study, we used focus groups and interviews to enable a comparison of the distinct learning cultures of medicine and music; the aim of this comparative approach was to reveal taken-for-granted assumptions inherent in medicine’s learning culture. We chose music as a comparator both for its shared features and its anticipated differences. Like medicine, music trains its learners to perform, engaging specialized skills in specific settings. We anticipated, however, that the process of learning to perform and the very nature of the performance goals in music were sufficiently distinct from medicine to provide meaningful insights. In our fourth study, we analysed focus group discussions and individual interviews related to the experience of receiving feedback in three learning cultures—medicine, music, and schoolteacher training—with the aim of developing an understanding of how learning culture influences the handling of feedback. Finally, in study 5, we interviewed medical students or practising doctors who had also had high-level training in music and sports—individuals who had travelled between learning cultures—to explore how individual and sociocultural influences on feedback interact.

## Results

1. As they participate in clinical work, medical learners can attend to a variety of sources of information, or *learning cues*, which shape their development. Feedback competes for learners’ attention with other learning cues, including role models, clinical outcomes, patient or family responses, and comparisons with peers. To win a learner’s attention and become influential, feedback must survive a critical judgement of its credibility [11].
2. Regulatory focus theory provides some insight into medical learners’ responses to feedback, particularly in circumstances where an individual’s regulatory focus can be readily determined. There are challenges in applying regulatory focus theory to real feedback scenarios, however. Clinical tasks often resist classification as activating *either* promotion focus *or* prevention focus for learners. Rather, a mixed regulatory focus is often present; learners may be motivated simultaneously by their aspirations around their burgeoning identity as doctors (promotion focus) and their desire to ensure safe task performance and avoid errors (prevention focus), complicating efforts to predict their responses to feedback. Furthermore, regulatory focus can change over time, as individuals reflect on and reframe tasks; with this change, there can be an evolution of the learner’s response to the feedback they receive. Finally, other factors, such as the perceived credibility of the source or content of the feedback, may trump regulatory focus in directing learners’ responses [12].
3. Our comparison of medicine’s learning culture with that of music revealed taken-for-granted assumptions that underpin medicine’s pedagogical approaches, and raised questions about these assumptions and their value for learning. While medicine values *learning by doing*, characterized by immersion in real clinical experiences, music values *learning by lesson*, characterized by methodical one-on-one instruction and individual practice. While medical learners aim for competence, music students aim instead for ever-better performance. While medical learners value their teachers for their clinical skills more than for their teaching abilities, the opposite is true in music, where teachers’ instructional skills are paramount. Self-assessment challenges learners in both cultures, but medical learners view self-assessment as a skill they can develop, while music students recognize that external feedback will always be required [13].
4. Although learners across cultures identify *credibility* and *constructiveness* as essential for feedback to be perceived as meaningful, the very definitions of credibility and constructiveness are distinct to each learning culture. Learning cultures vary considerably in how effectively they support the occurrence of feedback possessing these critical characteristics. The learning cultures of both teacher training and music, for example, embed opportunities for direct, thorough observation of learner performance into their pedagogies in a way that medicine does not; by firmly grounding their feedback in direct observation, these learning cultures strengthen its credibility [14].
5. There are both individually and culturally defined elements of the experience of learning and receiving feedback. Learners’ motivation and orientation toward feedback, as well as the value they place on certain fundamental feedback characteristics—specificity, timeliness, credibility, and actionability—appear stable across learning contexts. Learning culture modulates the impact of feedback by creating the conditions and opportunities for good feedback to occur and for learners to respond [15].

## Discussion

Integrating the findings from this series of studies, we were able to establish a more robust conceptual and theoretical understanding of feedback. We argue that neither the individual nor the sociocultural perspective on learning is fully adequate to account for the complexity of how feedback is experienced and how it acquires meaning. We propose instead a model for understanding feedback that considers both the individual learner and the learning culture as essential and inseparable elements of the process. Neither can be addressed in isolation.

Although some individual variability in feedback response is inevitable, good feedback must be available within a learning culture in order for its learning impact to be realized. If a culture routinely creates barriers to good feedback, as often occurs within medicine, learners may seek guidance elsewhere, with the end result being a gradual diminution in the value of feedback for learning within that culture. Learning culture contributes more than just the support and opportunity for meaningful feedback to occur, however; a learning culture’s norms and values also shape what counts as credible feedback and what feedback demands learners’ attention. Learning culture thus shapes the very definition of *meaningful feedback* for learners.

Our work offers direction for enabling feedback to reach its potential as a meaningful guide to learning within medical training. Medicine must remedy the features of its culture that compromise the credibility and constructiveness of its feedback. Teachers and learners would benefit from more consistent opportunities for the deliberate and direct observation of learner performance that underpins credible feedback. They would benefit from opportunities for longitudinal teacher-learner relationships to develop, within which constructive, action-oriented feedback can more comfortably and authentically be exchanged [16]. Medicine’s learning culture would do well to normalize critical feedback, as coaching cultures such as music and sports do, to define clear performance goals around which feedback can be built, and to ensure that the goals for learners and teachers align.

## Conclusion

The central contribution of this thesis is to direct the attention of medical educators towards two historically marginalized elements of the process by which feedback achieves impact: learner perceptions and learning culture. The work not only shines a light on these key pieces of the puzzle, but also offers concrete guidance for improving our current status quo. In order to become a feedback culture, medicine needs to commit to building the supports necessary to enable meaningful feedback to occur.
